# The RNA chaperone Hfq has a multifaceted role in *Edwardsiella ictaluri*

**DOI:** 10.3389/fcimb.2024.1394008

**Published:** 2024-07-19

**Authors:** Ali Akgul, Safak Kalindamar, Adef O. Kordon, Hossam Abdelhamed, Iman Ibrahim, Hasan C. Tekedar, Attila Karsi

**Affiliations:** Department of Comparative Biomedical Sciences, College of Veterinary Medicine, Mississippi State University, Mississippi State, MS, United States

**Keywords:** *Edwardsiella ictaluri*, ESC, Hfq, SRNAs, stress response, virulence

## Abstract

*Edwardsiella ictaluri* is a Gram-negative, facultative intracellular bacterium that causes enteric septicemia in catfish (ESC). The RNA chaperone Hfq (host factor for phage Qβ replication) facilitates gene regulation via small RNAs (sRNAs) in various pathogenic bacteria. Despite its significance in other bacterial species, the role of *hfq* in *E. ictaluri* remains unexplored. This study aimed to elucidate the role of *hfq* in *E. ictaluri* by creating an *hfq* mutant (*Ei*Δ*hfq*) through in-frame gene deletion and characterization. Our findings revealed that the Hfq protein is highly conserved within the genus *Edwardsiella*. The deletion of *hfq* resulted in a significantly reduced growth rate during the late exponential phase. Additionally, *Ei*Δ*hfq* displayed a diminished capacity for biofilm formation and exhibited increased motility. Under acidic and oxidative stress conditions, *Ei*Δ*hfq* demonstrated impaired growth, and we observed elevated *hfq* expression when subjected to *in vitro* and *in vivo* stress conditions. *Ei*Δ*hfq* exhibited reduced survival within catfish peritoneal macrophages, although it had no discernible effect on the adherence and invasion of epithelial cells. The infection model revealed that *hfq* is needed for bacterial persistence in catfish, and its absence caused significant virulence attenuation in catfish. Finally, the *Ei*Δ*hfq* vaccination completely protected catfish against subsequent *Ei*WT infection. In summary, these results underscore the pivotal role of *hfq* in *E. ictaluri*, affecting its growth, motility, biofilm formation, stress response, and virulence in macrophages and within catfish host.

## 1 Introduction

The intracellular Gram-negative bacterium *Edwardsiella ictaluri* is a leading cause of enteric septicemia in catfish (ESC), posing a significant economic threat to farm-raised catfish. *E. ictaluri* rapidly attaches to and penetrates the host mucosal membranes and can survive and replicate inside catfish neutrophils and macrophages (Ainsworth and Chen, 1990; Booth et al., 2009). Although certain virulence factors, such as flagella, outer membrane proteins, lipopolysaccharide, type III and VI secretion systems, and extracellular proteins, have been identified (Newton and Triche, 1993; Klesius and Sealey, 1996; Lawrence et al., 2001; Bader et al., 2004; Thune et al., 2007; Kalindamar et al., 2023), the full spectrum of *E. ictaluri*’s virulence mechanisms remains incompletely understood.

Challenges persist despite using antimicrobials, bacterins, and live attenuated vaccines (LAV) to control ESC. Feed-additive antibiotics like Aquaflor, Romet 30, and Terramycin face reduced efficacy due to the early-onset of reduced appetite in infected fish. Furthermore, their usage may contribute to antibiotic resistance in *E. ictaluri* strains (Tu et al., 2008; Erickson et al., 2024). Bacterins provide limited benefits, whereas LAVs can stimulate innate and cellular immunity (Ellis, 2001; Kordon et al., 2020, 2021). Thus, *E. ictaluri* LAVs provide adequate protection (Cooper et al., 1996; Lawrence et al., 1997; Klesius and Shoemaker, 1999; Thune et al., 1999; Wise et al., 2015). Our research group developed two novel *E. ictaluri* live attenuated strains (*Ei*Δ*evpB* and ESC-NDKL1), providing significant protection against ESC in fry and fingerling catfish (Nho et al., 2017; Abdelhamed et al., 2018).

Small RNAs (sRNAs) have been found in both prokaryotes and eukaryotes (Meibom et al., 2009), and they are involved in the regulation of metabolism and virulence mechanisms (Oliva et al., 2015). Bacterial sRNAs respond dynamically to environmental stress, modulating transcription, translation, and RNA degradation. sRNAs employ both cis- and trans-encoded base-pairing mechanisms. Cis-encoded sRNAs exhibit complete complementarity with their target mRNA, while trans-acting sRNAs can interact with multiple mRNA targets and often necessitate the involvement of the RNA chaperone protein Hfq. While Hfq is generally considered dispensable for cis-encoded sRNA regulation, exceptions exist, underscoring the intricate involvement of Hfq in the regulatory dynamics of both cis- and trans-encoded sRNAs (Ellis et al., 2015; Brantl and Muller, 2021).

In Gram-negative bacteria, Hfq is essential for sRNAs’ activity and/or stability (Soper et al., 2010; Thomason and Storz, 2010; Wilton et al., 2015). The *hfq* gene is prevalent in half of all sequenced bacterial species (Sun et al., 2002). Beyond its role in virulence mechanisms, *hfq* governs quorum sensing, stress resistance, and various cellular functions, including osmotic stress, ethanol response, temperature shifts, and iron starvation (Fantappiè et al., 2009; Liu et al., 2011). Its impact extends to cell membranes, type III secretion system (T3SS), flagella, fimbria, biofilms, and overall bacterial fitness (Sittka et al., 2007; Chao and Vogel, 2010; Rempe et al., 2012; Caldelari et al., 2013; Cui et al., 2013; Meng et al., 2013; Kakoschke et al., 2014).

Studies on intracellular bacterial pathogens have underscored *hfq*’s significance in bacterial virulence (Chao and Vogel, 2010; Feliciano et al., 2016). This RNA chaperone serves as a virulence factor in diverse pathogenic bacteria, including *Brucella abortus, Legionella pneumophila, Salmonella* Typhimurium*, and Yersinia enterocolitica* (Zeng et al., 2013; Hu et al., 2014). *hfq* mutants display attenuated virulence in various animal models. For example, the *hfq* mutant of *S.* Typhimurium was attenuated in mice and showed reduced survival in macrophages (Sittka et al., 2007). The *hfq* mutant of *S.* Enteritidis exhibits significantly reduced virulence in chickens (Meng et al., 2013). Furthermore, in *Y. enterocolitica* and *B. melitensis*, *hfq* affected the metabolism, stress response, and production of virulence factors (Cui et al., 2013; Kakoschke et al., 2014). Similarly, *hfq* played a crucial role in the virulence of *Neisseria meningitides*, as shown in *ex vivo* and *in vivo* infection models (Fantappiè et al., 2009). The *hfq* mutant was found to be a LAV candidate against *B. melitensis* infection (Zhang et al., 2013). In *E. tarda*, the *hfq* mutant was attenuated in both macrophages and fish (Hu et al., 2014).

Considering the well-established role of sRNAs in regulating bacterial virulence, investigating the bacterial RNA-binding protein Hfq offers an intriguing approach to unraveling its involvement in *E. ictaluri* virulence. Additionally, the resulting attenuated strains could serve as valuable tools for probing the catfish immune system, potentially offering protection against *E. ictaluri* infections.

## 2 Materials and methods

### 2.1 Bacteria, plasmids, and media

The bacterial strains and plasmids used in this study are detailed in **Table 1**. The wild-type *E. ictaluri* strain 93-146 (*Ei*WT) was cultivated at 30°C in brain-heart infusion (BHI) broth for 16 h or on agar plates for 2 days. *Escherichia coli* CC118λ*pir* and BW19851 strains were propagated in Lysogeny broth (LB) or on Lysogeny agar (LA) at 37°C for 16 h. Culture media were supplemented with ampicillin (100 mg/ml), colistin (12.5 mg/ml), sucrose (5%), and mannitol (0.35%) when required.

**Table 1:** Bacterial strains and plasmids.

Strains and plasmids	Characteristics	Reference or source
*Edwardsiella ictaluri*		
93-146	Wild-type; pEI1; pEI2; *Col^r^*	(Lawrence et al., 1997)
* Ei*Δ*hfq*	93-146 derivative; pEI1; pEI2; *Col^r^*, Δ*hfq*	This study
* Ei*Δ*hfq*+p*Eihfq*	*Ei*Δ*hfq*, *hfq*	This study
*Escherichia coli*		
CC118λ*pir*	Δ*(ara-leu); araD;* Δ*lacX74; galE; galK; phoA20; thi-1; rpsE; rpoB; argE(Am); recAl; λpirR6K*	(Herrero et al., 1990)
SM10λ*pir*	*thi; thr; leu; tonA; lacY; supE; recA;::RP4-2-Tc::Mu; Kan^r^; λpirR6K*	(Miller and Mekalanos, 1988)
BW19851	*RP4-2 (Km::Tn7, Tc::Mu-1), DuidA3::pir+, recA1, endA1, thi-1, hsdR17, creC510*	(Metcalf et al., 1994)
DH5α	*fhuA2Δ(argF-lacZ)U169 phoA glnV44 Φ80Δ(lacZ)M15 gyrA96 recA1 relA1 endA1 thi-1 hsdR17*	(Taylor et al., 1993)
Plasmids		
pMEG375	8142 bp, *Amp^r^*, *Kan^r^*, *lac*Z, R6K *ori*, *mob incP*, *sacR sacB*	(Dozois et al., 2003)
p*Ei*Δ*hfq*	10230 bp, pMEG-375, Δ*hfq*	This study
pBBR1MCS-4	4950 bp, *Amp^r^*, *mob*, *rep*, *lacI*, *lacZ* P*_lac_*	(Kovach et al., 1995)
p*Eihfq*	5259 bp, pBBR1MCS-4, *hfq*	This study
pAK*gfplux*1	11547 bp, pBBR1MCS-4, *gfp*, *luxCDABE*	(Karsi and Lawrence, 2007)

### 2.2 Comparative analysis of the Hfq protein sequences

The comparative analysis involved aligning the Hfq protein sequence of *E. ictaluri* strain 93-146 with members of the *Enterobacteriaceae* family. The analysis included *E. ictaluri* strains LADL11-100 and LADL11-194, *E. anguillarum* ET080813, *E. hoshinae* ATCC 35051, *E. piscicida* strains FL6-60, C07-087, RSB1309, JF1305, and EIB202, as well as *E. tarda* strains ATCC 15947, ATCC 23685, and FL95-01. Members from other bacterial families, such as *Klebsiella pneumoniae subsp. pneumoniae* NTUH-K2044, *Salmonella enterica* serovar Typhimurium str. LT2, *Yersinia enterocolitica subsp. enterocolitica* 8081, and *Shigella flexneri* 2a str. 301, were also included. Additionally, representatives from α–Proteobacteria (*Brucella abortus* bv.1 str. 9-941 and *Agrobacterium radiobacter* K84) and β–Proteobacteria (*Bordetella pertussis* Tohama I, *Burkholderia cenocepacia* AU 1054, and *Neisseria meningitidis* MC58) were utilized. The phylogenetic tree was constructed using MEGA v11.0 software, utilizing the maximum likelihood method with 500 bootstraps (Kumar et al., 2016). Hfq protein sequences were aligned using CLC Genomics Workbench 6.5.1 from CLC Bio.

### 2.3 In-frame deletion of the *hfq* gene

**Figure 1:**
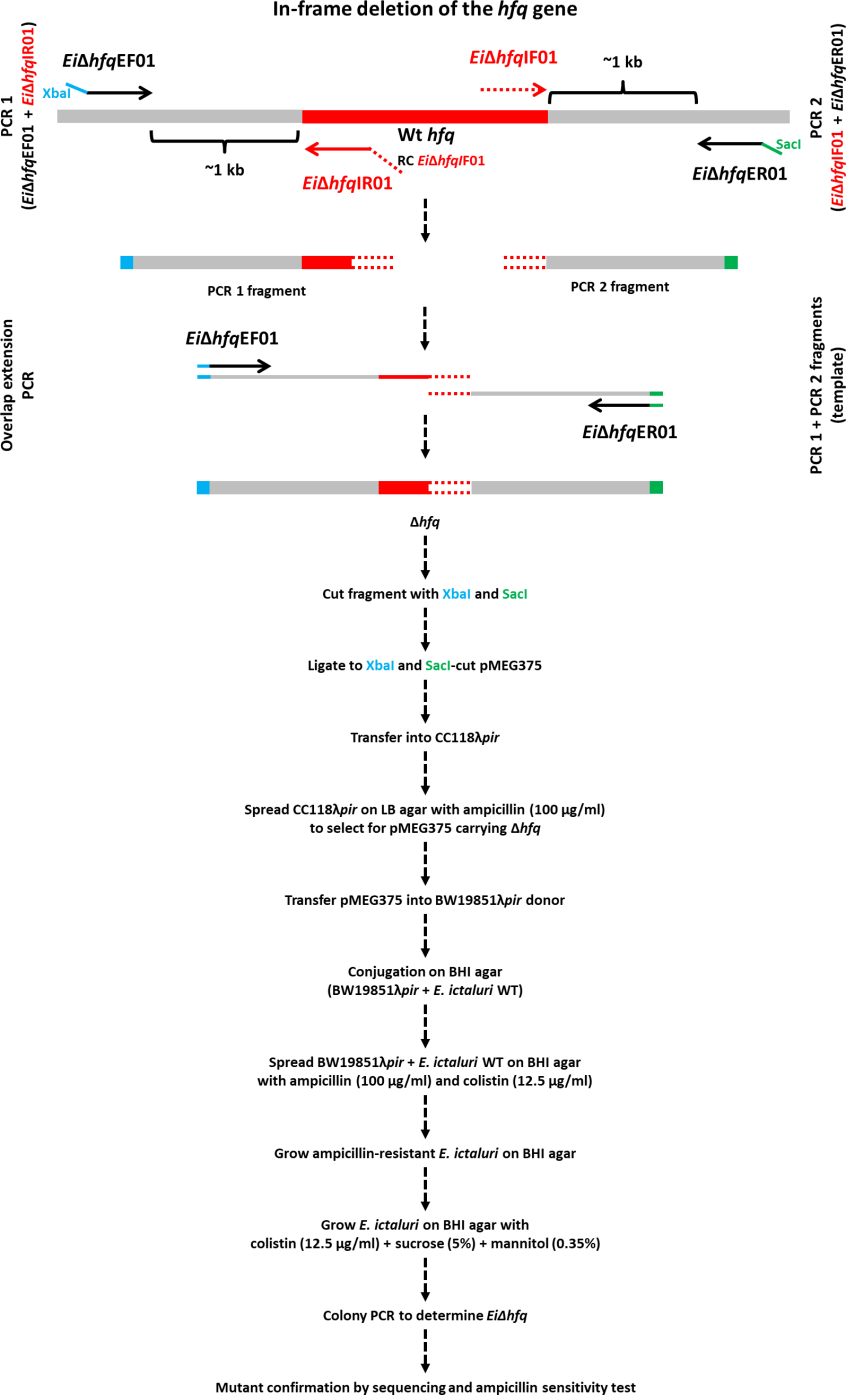
In-frame deletion of the *hfq* gene. The *hfq* gene is represented by a red band, and grey bands represent upstream and downstream regions. The blue and green tails at the end of *Ei*Δ*hfq*EF01 and *Ei*Δ*hfq*ER01 primers indicate XbaI and SacI restriction enzymes, respectively. The red tail at the end of *Ei*Δ*hfq*IR01 primer represents the reverse complement of *Ei*Δ*hfq*IF01 primer. The overlapping region is shown in a red round dotted band.

The nucleotide sequence otpf *hfq* (NT01EI_RS01835) was retrieved from the *E. ictaluri* strain 93-146 genome (GenBank accession: CP001600) (Williams et al., 2012), and the process of *hfq* mutant construction was illustrated in **Figure 1**. For the targeted in-frame deletion of the *hfq* gene, a set of two external and two internal primers was designed (**Table 2**). Genomic DNA from *Ei*WT was extracted using the Wizard Genomic DNA Kit (Promega), and this isolated DNA served as the template for subsequent PCR amplifications.

**Table 2:** Primers used for in-frame deletion, complementation, and expression analysis.

Primers	Sequence (5’ → 3’)^a^	Purpose	RE
*Ei*Δ*hfq*EF01	at**tctaga**GTGGAGATCATCAGCGTGGAT	Mutant construction	XbaI
*Ei*Δ*hfq*IR01	tacgccttattcagcgtcatcTTGTAAAGATTGCCCCTTAGC	Mutant construction	
*Ei*Δ*hfq*IF01	GATGACGCTGAATAAGGCGTA	Mutant construction	
*Ei*Δ*hfq*ER01	at**gagctc**CCAGCAGTACCGGGATCTCAT	Mutant construction	SacI
*Ei*Δ*hfq*F01S	TGTTAGTGCATCGCTTGACTG	Sequencing	
*Ei*Δ*hfq*F01cmp	at**ggtacc**ATGGCTAAGGGGCAATCTTTA	Complementation	
*Ei*Δ*hfq*R01cmp	at**gagctc**TTATTCAGCGTCATCGCTGCC	Complementation	
Ei*hfq*F01	GCTAAGGGGCAATCTTTACAAG	qRT-PCR	
Ei*hfq*R01	CGGTAGAGATCGCGTGTTTG	qRT-PCR	
Ei16sRNAF01	AGAGTTTGATCATGGCTCAG	qRT-PCR	
Ei16sRNAR01	GGTTACCTTGTTACGACTT	qRT-PCR	

a Bold letters show restriction enzyme (RE) recognition sequences added to primers. Two bases (at) were added before RE sequences to increase enzyme efficiency. The underlined sequence in EiΔhfqIR01 primer indicates a reverse complement of EiΔhfqIF01 primer, which forms an overlapping region for gene deletion by overlap extension PCR.

The amplified upstream and downstream fragments underwent gel extraction using the QIAquick Gel Extraction Kit (Qiagen). These fragments were then mixed in equal proportions to serve as a template for the subsequent overlap extension PCR. The resulting in-frame deletion fragment was digested with XbaI and SacI restriction enzymes (New England Biolabs) and cleaned up.

The suicide plasmid pMEG375 was purified from an overnight *E. coli* culture by a QIAprep Spin Miniprep Kit (Qiagen) and cut with the same enzymes for cloning. The purified PCR product with in-frame deletion was ligated into the pMEG375 vector using T4 DNA Ligase (New England Biolabs) at 16°C overnight. *E. coli* CC118λ*pir* was transformed by electroporation and plated on LB agar with ampicillin. Plasmid DNAs were prepared from the colonies, and cloning success was confirmed by plasmid size, cloned fragment size, and sequencing.

The correct plasmid, named p*Ei*Δ*hfq*, was transferred into *E. coli* BW19851λ*pir* by chemical transformation and mobilized into *Ei*WT by conjugation. After conjugation, plasmid integration was achieved by ampicillin selection. Then, ampicillin-resistant colonies were propagated on BHI agar to allow for the second allelic exchange. After this step, colonies were streaked on BHI plates with 5% sucrose, 0.35% mannitol, and colistin to select for loss of pMEG375. The final confirmation of plasmid loss was achieved by testing *E. ictaluri* colonies for ampicillin sensitivity. PCR and sequencing procedures validated the successful deletion of *hfq* in *E. ictaluri*, and the resultant mutant strain was designated as *Ei*Δ*hfq*.

### 2.4 Complementation of *Ei*Δ*hfq*

For complementation of *Ei*Δ*hfq*, a wild-type copy of the *hfq* gene was amplified from *Ei*WT genomic DNA using the primers *Eihfq*F01cmp and *Eihfq*R01cmp, which contained KpnI and SacI restriction sites, respectively (**Table 2**). After digestion with both enzymes and clean-up, the amplified fragment was cloned into KpnI- and SacI-digested broad host range pBBR1MCS4 (Kovach et al., 1995). *E. coli* DH5α was transformed with 1 µl of the ligation mixture, and *E. coli* transformants containing p*Eihfq* were identified by colony PCR, and the *hfq* sequence in p*Eihfq* was confirmed by sequencing. Then, p*Eihfq* was transformed into *Ei*Δ*hfq* by conjugation, and complemented colonies (*Ei*Δ*hfq*+p*Eihfq*) were determined on BHI agar with ampicillin and colistin selection.

### 2.5 Construction of bioluminescent *Ei*Δ*hfq*

The pAK*gfplux*1 plasmid (Karsi and Lawrence, 2007) was transferred into *Ei*Δ*hfq* by conjugal mating to construct bioluminescent *Ei*Δ*hfq*. Briefly, *E. coli* SM10λ*pir* carrying pAK*gfplux*1 was used as a donor strain to transfer the plasmid into recipient *Ei*WT. Overnight cultures of donor and recipient were mixed at a 1:2 donor: recipient ratio. The mixture pellet was transferred onto sterile 0.45 µM filter paper on a BHI agar plate and incubated at 30°C for 24 h. Bacteria on filter paper were dissolved in BHI broth with ampicillin and colistin and then spread on BHI plates containing ampicillin and colistin. After incubation at 30°C for 24-48 h, ampicillin-resistant *Ei*WT colonies carrying pAK*gfplux*1 appeared on plates, and their bioluminescence was verified by IVIS Lumina XRMS in Vivo Imaging System Series III (PerkinElmer).

### 2.6 Growth kinetics of *Ei*Δ*hfq*

*Ei*WT, *Ei*Δ*hfq*, and complemented *Ei*Δ*hfq* strains were streaked on BHI agar containing colistin. A single colony from BHI agar was inoculated into 5 ml of BHI broth with colistin. When optical density at 600 nm (OD_600_) reached 0.5, bacterial culture was diluted 50 times with 50 ml of BHI broth with colistin in a 250 ml flask, which was incubated in a rotary shaker at 200 rpm at 30°C. OD_600_ values were measured for 60 h by sampling bacterial cultures every 6 h. At 12, 24, and 48 h, serially diluted cultures were spread on BHI agar for colony counting. Four replicates were used for each strain.

### 2.7 Biofilm formation

Biofilm formation in *Ei*Δ*hfq*, *Ei*WT, and complement strains was assessed using a protocol described by other authors (Cai and Arias, 2017). Briefly, 10 μl of overnight cultures were inoculated in 90 μl BHI in flat-bottom 96-well microtiter plates, and cultures were grown without disruption at 30°C for 48 h to allow biofilm formation. Following removal of planktonic cells, the wells were stained with crystal violet (150 ul, 1%) for 20 min. Then, crystal violet was removed by washing, and the remaining dye was dissolved in ethanol (96%) and quantified by measuring the OD_600_ values.

### 2.8 Motility test and scanning electron microscopy

For motility assay, bacterial strains were grown in BHI broth until the late-log phase (OD_600_ = 1.0), then 1 μl culture was spotted onto BHI plates containing colistin, ampicillin, 1 mM arabinose, 0.3% agar (swimming motility plate), and 0.5% agar (swarming motility plate). The plates were incubated at 30°C for 24, 48, and 72 h to observe bacterial motility zones.

For SEM analysis, aliquots of a 2 mL suspension of logarithmic-phase bacteria were incubated on sterile poly-L-lysine-coated coverslips in a sterile polystyrene 6-well plate at 25°C for 24 h to allow adhesion to occur. After the 24-h incubation, non-adherent bacteria were removed by pipetting and washed with 3 mL of sterile distilled water. The cells were fixed with 0.5% Karnovsky’s fixative in 0.1 M sodium cacodylate buffer, pH 7.2, for a minimum of 24 h. Subsequently, the cells were rinsed, postfixed in 2% osmium tetroxide for 1 h, and then dehydrated with a series of ethanol dilutions (35%, 50%, 70%, 95%, and 100%) for 15 minutes each. The adhered cells were transferred to a graded mixture of hexamethyldisilazane (HMDS) and ethanol (25%, 50%, 75%), followed by 100% HMDS for 1 h. This was followed by overnight air-drying of the samples, which were immediately coated with 45 nm of platinum in an EMS-150T ES sputter coating operation and examined using a JEOL JSM 6500 scanning electron microscope.

### 2.9 Survival of *Ei*Δ*hfq* in acidic and oxidative stress

After overnight growth, OD_600_ values were measured to adjust culture volumes. Subsequently, 5 µl of bacteria were inoculated into 195 µl of BHI broth with ampicillin and colistin in 96-well black plates. The medium was modified to create acidic (pH=5.5) and oxidative stress conditions (3 mM H_2_O_2_). Untreated BHI was used as control. A Cytation 5 Cell Imaging Multimode Reader (BioTek) captured bioluminescence for 3 h at 30°C. Each strain had four replicates, and the experiment was repeated three times.

### 2.10 Expression of *hfq* under *in vitro* and *in vivo* stresses

A single *Ei*WT colony was inoculated into 5 ml of BHI broth, followed by 16-18 h incubation at 30°C with shaking at 200 rpm. For each group, 40 ml of BHI broth was inoculated and grown until OD_600_ reached 0.4. Then, each culture was divided into four aliquots of 10 ml, and bacteria were harvested by centrifugation at 6,000 x *g* for 15 min. The supernatant was removed, and cells were resuspended in 10 ml of fresh BHI supplemented with 1.5 mM H_2_O_2_ (0.05%) and BHI broth acidified with a 6 N HCl (pH=4). Cultures were incubated by shaking at 180 rpm at 30°C for 30 min. The bacteria were harvested, the supernatant was removed, and RNAlater solution was added to the pellets, which were stored for a week at -20°C until RNA isolation.

For serum treatment, *Ei*WT was exposed to naïve channel catfish serum, with heat-inactivated catfish serum used as a control. Each treatment comprised four biological replicates. *E. ictaluri* cultures underwent three washes using 1.25 ml of cell wash buffer (10 mM Tris-HCl and 5 mM magnesium acetate). Subsequently, normal and heat-inactivated serum (1.25 ml) was added to the *E. ictaluri* pellet, mixed, and incubated for 30 min at 30°C. Following incubation, the serum-bacteria mixture was used for total RNA isolation.

For *in vivo* stress, eighteen-month-old specific pathogen-free channel catfish fingerlings were stocked into two tanks at a rate of 6 fish/tank. After one week of acclimation, fish were anesthetized in water containing 100 mg/L MS-222 (Sigma) and injected with bioluminescent *Ei*WT (approximately 1x10^4^ CFU) in 100 µl PBS. Negative control was injected with 100 µl PBS. After 30 h of bioluminescent imaging, fish were euthanized with 400 mg/L MS-222 (Sigma), and head kidney, liver, and spleen were collected in tubes with RNA later solution.

### 2.11 Total RNA extraction, cDNA synthesis, and qRT-PCR

The extraction of total RNA was performed using the RNeasy Protect Bacteria Mini Kit (Qiagen), following the manufacturer’s guidelines. DNase treatment was carried out using the RNase-Free DNase Set (Qiagen) to eliminate potential DNA contamination. The quality and concentration of the isolated total RNA were evaluated using NanoDrop 1000 (Thermo Fisher). Subsequently, 1 µg of total RNA was converted to cDNA utilizing the Maxima First Strand cDNA Synthesis Kit for RT-qPCR (Thermo Fisher) following the user manual.

Quantitative real-time PCR (qRT-PCR) was performed using DyNAmo SYBR Green qPCR Kit (Thermo Fisher) and an Mx3005P qPCR System (Agilent). PCR reactions contained 10 µl SYBR Green 2X mix, 0.2 µM each of forward and reverse primers, and 1 µl of 100X diluted cDNA (**Table 2**). The PCR was set to initial denaturation at 95°C for 3 min, 45 cycles of denaturation at 95°C for 15 s, annealing at 60°C for 30 s, and extension at 72°C for 30 s, and a final extension at 72°C for 3 min. At the end of the PCR, a melting curve program was run from 60°C to 95°C with a 0.5°C increase every 15 s. 16S RNA gene was used as an internal control (**Table 2**). A sample from unstressed conditions was set as a calibrator in each experiment, except heat-treated serum was used as a calibrator against normal serum. Relative expression rates were calculated by the threshold cycle changes in the sample and calibrator. The ΔΔCt method was used to calculate the expression level of related genes (Livak and Schmittgen, 2001). All expression values were normalized against 16S rRNA. ΔΔCt was calculated by ΔΔCt = ΔCt (stress condition) - ΔCt (non-stress condition), where ΔCt is the normalized signal level in a sample (ΔCt = Ct of target gene – Ct of reference gene).

### 2.12 Survival in peritoneal macrophages

The bacterial killing assay was performed as previously described with minor modifications (Kalindamar et al., 2023). Briefly, four days after squalene injection, peritoneal macrophages were collected from five one-year-old channel catfish (250-300 g) by injecting 10 ml cold phosphate-buffered saline (PBS) to the peritoneal cavity and harvesting macrophages by using a three-way valve. Harvested cells were washed with PBS three times, and resuspended in channel catfish macrophage medium (CCMM), including RPMI [(RPMI 1640 sans phenol red & L-glutamine, Lonza) containing 1x glutamine substitute (GlutaMAX –I CTS, Gibco)], 15 mM HEPES buffer (GIBCO), in 0.18% sodium bicarbonate solution (GIBCO), 0.05 mM 2-beta-mercaptoethanol (all from Sigma), and 5% heat-inactivated (HI) channel catfish serum.

Peritoneal macrophages and bioluminescent *Ei*WT and *Ei*Δ*hfq* were suspended in a 1:1 ratio and placed in a 96-well black plate as four replicates. A negative control group without bacteria was also included in the plate. Cells and bacteria were compacted by centrifugation of the plate at 1500 rpm for 5 min at 24°C. Then, the plate was incubated with CCMM for 1 h at 30°C and centrifuged at 2000 rpm for 7-10 min. After removing the supernatant, CCMM containing 100 µg/ml gentamicin was added to the plate, which was incubated for 1 h at 30°C to kill non-phagocyted bacteria. Extracellular bacteria were removed by washing the wells 3 times with PBS. Next, the cell-bacteria mixture was incubated with CCMM containing 10 µg/ml gentamicin for 48 h with 5% CO_2_ at 30°C, and Cytation 5 Cell Imaging Multimode Reader (BioTek) was used to acquire bioluminescence emitted by the surviving bacteria inside the catfish peritoneal macrophages.

### 2.13 Cytospin and light microscopy

After incubating the cell-bacteria mixture with CCMM containing 10 µg/ml gentamicin, as described above, cells were harvested at 4 h post-challenge and washed with PBS. Subsequently, cytospin preparations were applied at 500 rpm for 1 min using a Cyto-Tek centrifuge. Following this, all samples underwent fixation and staining with Wright’s stains (Hemacolor, Merck), following previously published procedures (do Vale et al., 2002). Finally, the samples were analyzed and photographed using an Olympus BX microscope (Olympus U-TV1 X) and Infinity software.

### 2.14 Attachment and invasion in epithelial cells

Attachment and invasion assay was performed as previously described with minor modifications (Kalindamar et al., 2020). Channel catfish ovary (CCO) cells were suspended in DMEM medium (Sigma) with 10% fetal bovine serum and 4mM L-glutamine at the final concentration of 1x10^7^ cells ml^-1^ and placed in a 24-well plate. The exact number of bioluminescent *Ei*WT and *Ei*Δ*hfq* were added to the plate to achieve 1:1 multiplicity of infection. Each plate contained four biological replicates and a negative control group without bacteria. CCO and bacteria suspension were incubated 1 h at 28°C to allow bacterial attachment to cells. Following the incubation, samples were washed three times with PBS. Then, DMEM medium, including 100 µg/ml gentamicin, was used to kill non-invasive *Ei*WT and *Ei*Δ*hfq.* Finally, the plate was incubated for another h at 28°C to determine bacterial invasion. Bioluminescence was captured by IVIS Lumina XRMS in Vivo Imaging System Series III and quantified from images by Living Image Software v 4.2.

### 2.15 Bioluminescent imaging in live catfish

Eight specific-pathogen-free (SPF) channel catfish fingerlings obtained from the MSU-CVM Hatchery (12.72 ± 5.47 cm, 24.95 ± 1.00 g) were stocked into two 50-liter tanks (four fish each) and acclimated for one week at 28°C. After lowering the tank water level to 10 liters, 100 ml cultures of *Ei*Δ*hfq* (treatment) and *Ei*WT (control) were added to each tank for the immersion challenge (final dose of 5x10^7^ CFU/ml water). After 1 h, the water flow was restored. Infected catfish were anesthetized in water containing 100 mg/L MS222 (Sigma) and immediately placed into the photon collection chamber of IVIS Lumina XRMS in Vivo Imaging System Series III for image capture. The exposure time was set to one minute to collect photon emissions from the whole fish body. After imaging, fish were placed in well-aerated water for recovery. Bioluminescent imaging was conducted at 0, 6, 12, and 24 h post-infection and subsequent daily intervals until 336 h. Living Image Software v 4.2 was used to quantify bioluminescence from fish images.

### 2.16 Virulence and efficacy of *Ei*Δ*hfq* in catfish

Virulence/vaccination and efficacy experiments were conducted as described in our earlier work (Karsi et al., 2009). Briefly, 500 specific-pathogen-free (SPF) channel catfish fingerlings obtained from the MSU-CVM Hatchery (3.81 ± 0.80 cm, 10.544 ± 0.99 g) were stocked into 20 tanks at a rate of 25 fish/tank and acclimated for one week at 28°C. Chlorine, dissolved oxygen, and temperature were monitored daily. Tanks were randomly assigned to five groups, with four tanks in each: *Ei*Δ*hfq* (vaccination), *Ei*Δ*hfq*+p*Eihfq* (complement), *Ei*WT (positive control), BHI (sham control), and negative control. Immersion vaccination was applied by lowering the water in each tank to 10 liters and adding 100 ml of bacterial culture (final dose of 2.1x10^7^ CFU/ml water). After 1 h, water flow (1-1/min) was restored to each tank. Mortalities were recorded daily for 21 days, and the percent mortalities were calculated for each group. To assess the *Ei*Δ*hfq* as a possible vaccine candidate, all fish that survived the *Ei*Δ*hfq* vaccination were re-challenged with *Ei*WT (final dose of 2.8x10^7^ CFU/ml water) 21 days post-vaccination as described above. Fish mortalities were recorded daily for 14 days.

### 2.17 Statistical analysis

Statistical analysis was conducted using one-way ANOVA and two-way ANOVA procedures with Tukey’s test in SAS for Windows 9.4 (SAS Institute, Inc., Cary, NC) to determine the significance of differences between means of treatments or groups. The predetermined significance level for all tests was set at *p* < 0.05. Photon emissions were log_10_ transformed to enhance normality, and a two-independent-samples t-test was employed for symmetrical variable comparison between *Ei*Δ*hfq* and *Ei*WT.

## 3 Results

### 3.1 Hfq protein is conserved in the genus *Edwardsiella*

**Figure 2:**
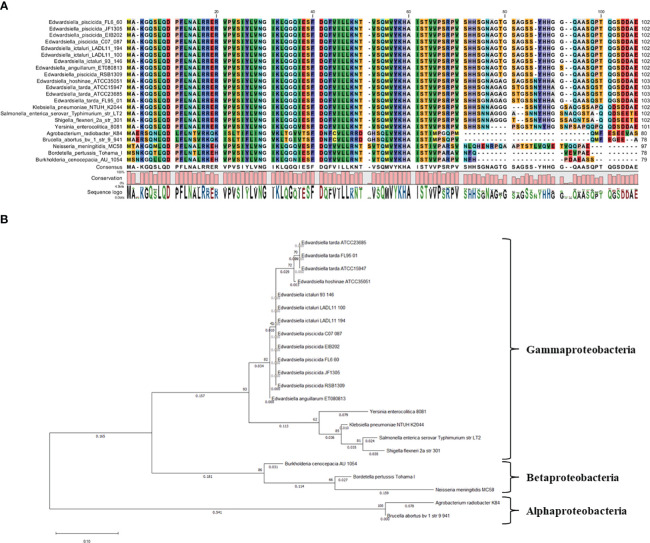
**(A)** alignment of Hfq proteins and **(B)** phylogenetic tree.

We identified a single functional *hfq* gene in the genome of *E. ictaluri* strain 93-146. The average size of the Hfq protein in the *Edwardsiella* genus is 102-103 amino acids (aa), while the Hfq protein’s average size in α- and β-Proteobacteria ranged between 78 and 97 aa (**Figure 2A**). Phylogenetic analysis revealed its conservation within *Enterobacteriaceae*, with a closer similarity to *α*-Proteobacteria than to *β*-Proteobacteria (**Figure 2B**).

### 3.2 *Ei*Δ*hfq* showed decreased growth and viability in the late exponential phase

**Figure 3:**
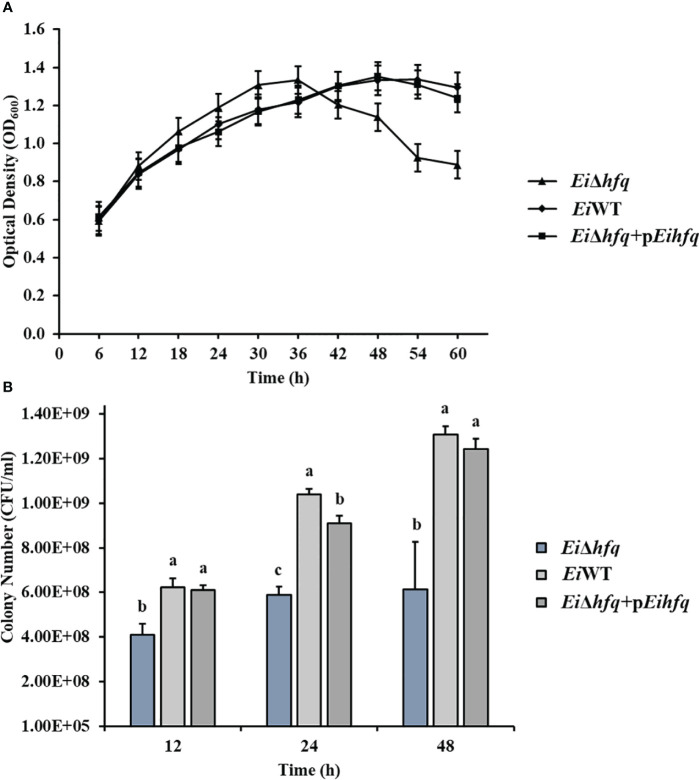
The growth kinetics of *Ei*Δ*hfq*, *Ei*WT, and complemented (*Ei*Δ*hfq*+p*Eihfq*) strains were evaluated under different conditions. **(A)** Optical density at 600 nm (OD_600_) was measured over 60 h at 30°C using a shaker incubator in Brain Heart Infusion (BHI) broth. The OD_600_ values provide insights into the bacterial growth trends over time. **(B)** Colony forming units per milliliter (CFU/ml) were determined during bacterial growth at 12, 24, and 48 h The cultures were incubated in a 30°C shaker incubator in BHI broth. Serial dilutions were performed, and colonies were counted after plating on BHI agar plates. This method allows for quantifying viable bacterial cells at different time points. Small letters indicate statistical differences between treatments (*p* < 0.05).

To assess the influence of *hfq* on *E. ictaluri* growth, we cultured *Ei*Δ*hfq*, *Ei*WT, and complement strains in broth and agar media. Although the initial growth stages showed comparable patterns across all three strains, a distinct decline in the growth of *Ei*Δ*hfq* became evident in the later stages (**Figure 3A**). Colony counts unveiled a significant reduction in the number of *Ei*Δ*hfq* colonies in comparison to *Ei*WT and complemented strains (*p* < 0.05) (**Figure 3B**).

### 3.3 Deletion of *hfq* affects biofilm formation

**Figure 4:**
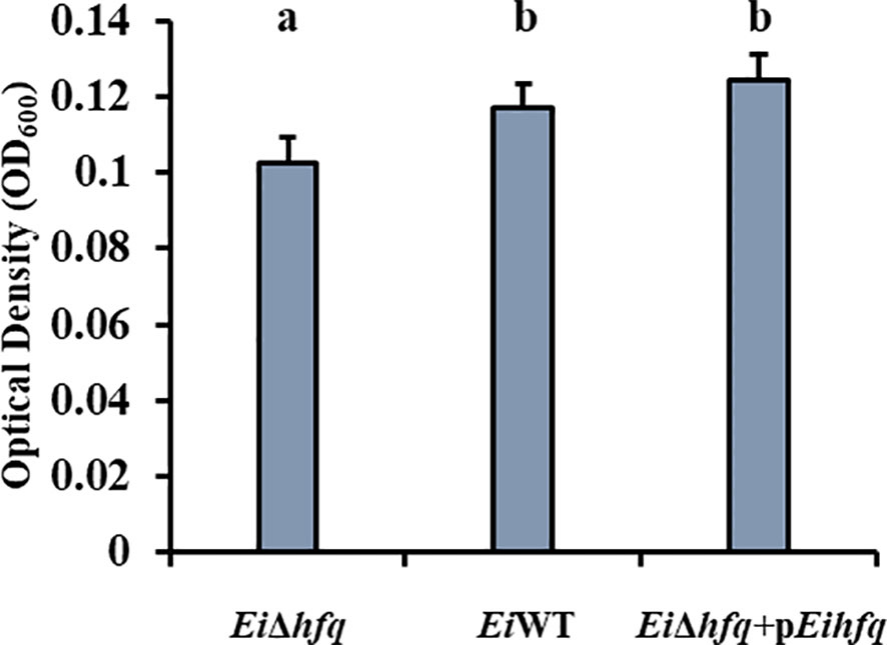
Biofilm formation of *Ei*Δ*hfq, Ei*WT, and complement strains at 48 h. Small letters indicate statistical differences between treatments (*p* < 0.05).

*Ei*Δ*hfq* formed significantly less biofilm compared to *Ei*WT and complement strains, while no significant differences were observed between *Ei*WT and complement strains at 48 h (*p* < 0.05) (**Figure 4**).

### 3.4 Deletion of *hfq* affects motility with little effect on flagella

**Figure 5:**
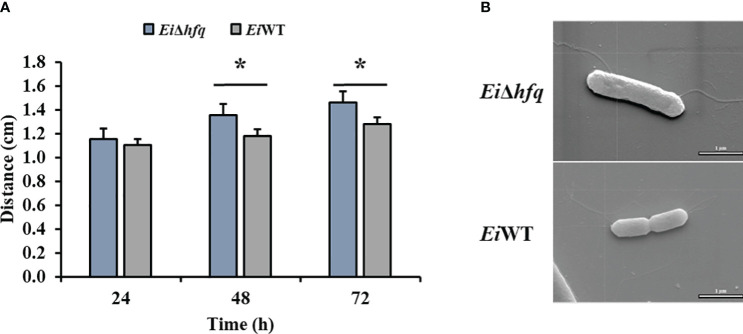
Motility and SEM. **(A)** The swimming zones of *Ei*Δ*hfq* and *Ei*WT were determined at 24, 48, and 72 h by measuring diameters (cm). Statistical significance is shown by an asterisk (*p* < 0.05). **(B)** SEM photomicrograph of representaive *Ei*Δ*hfq* and *Ei*WT strains with flagellar filaments (scale bar is 1 µm).

*Ei*Δ*hfq* exhibited swimming motility comparable to *Ei*WT at 24 h. However, the motility of *Ei*Δ*hfq* was significantly higher than that of *Ei*WT at 48 h and 72 h (**Figure 5A**). SEM imaging of *Ei*Δ*hfq* and *Ei*WT revealed the presence of flagella in both strains. There were numerous thin and few dense lateral flagellar filaments in *Ei*WT and *Ei*Δ*hfq*, respectively (**Figure 5B**).

### 3.5 *hfq* is essential for survival under acidic and oxidative stresses

**Figure 6:**
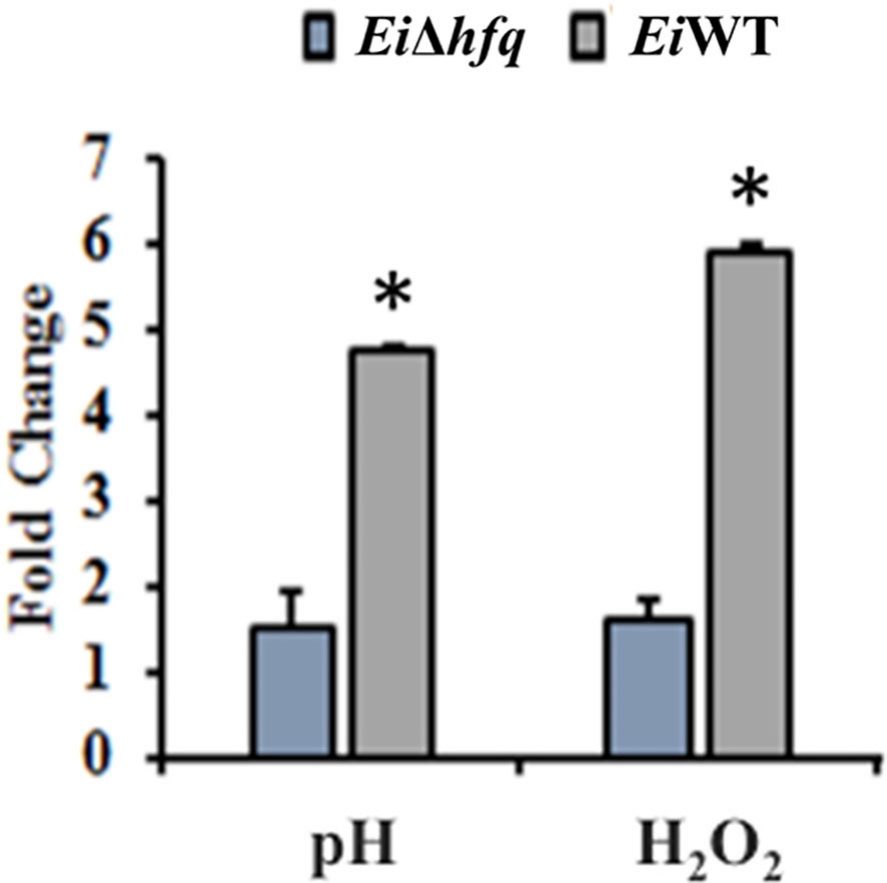
Fold change of bioluminescence in acidic (pH 5.5) and oxidative (3 mM H_2_O_2_) stresses. Untreated BHI was used as a control to calculate fold changes in each treatment. The (*) symbol indicates a significant difference between treatments (*p* < 0.01). The data are representative of three independent experiments.

Exposure of *Ei*Δ*hfq* to acidic and oxidative stresses resulted in a three- to four-fold reduction in its bioluminescence compared to *Ei*WT (**Figure 6**), indicating the mutant’s diminished capability to cope with these stresses.

### 3.6 The *hfq* gene is highly expressed *under in vitro* and *in vivo* stress conditions

**Figure 7:**
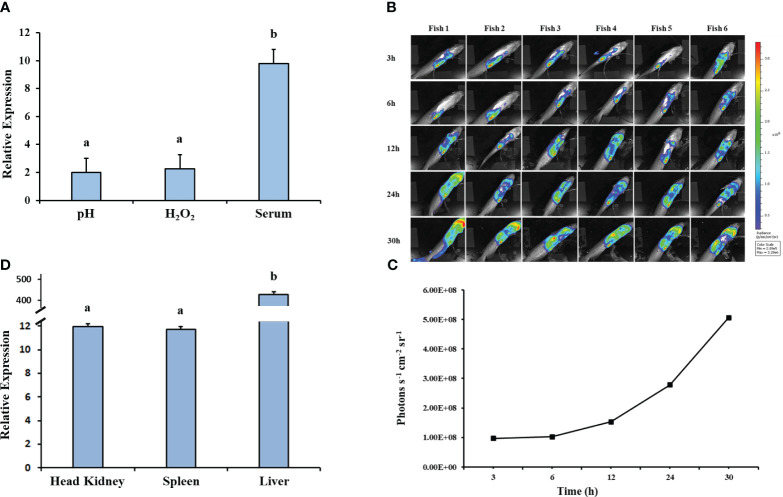
Gene expression analysis of *hfq*. **(A)** Relative gene expression values of *hfq* in *E. ictaluri* exposed to *in vitro* stress factors: low pH (4), H_2_O_2_ (1.5mM), and channel catfish serum. The gene expression was relative to the BHI growth of *E. ictaluri*. Small letters indicate statistical differences between treatments (*p* < 0.05). **(B)** Bioluminescent imaging of *E. ictaluri* in six live catfish after intraperitoneal injection. The imaging times are marked on the left, and the color scale on the right shows photon emission intensity from low (blue) to high (red). **(C)** Numerical values of photon emissions at each time point. Head kidney, spleen, and liver tissues were collected after imaging catfish at 30 h, and **(D)** *hfq* expression relative to the BHI-grown *E. ictaluri* was determined. The expression values were normalized by 16S rRNA. Small letters indicate statistical differences between treatments (*p* < 0.05).

The relative expression of *hfq* in *Ei*WT increased significantly under *in vitro* stresses (pH 4 and 1.5 mM H_2_O_2_) (**Figure 7A**). *Ei*WT demonstrated replication in fish over time (**Figures 7B, C**), and growth in the head kidney, spleen, and liver environments induced *hfq* expression, particularly in the spleen (**Figure 7D**).

### 3.7 Deletion of *hfq* resulted in increased bacterial killing in peritoneal macrophages

**Figure 8:**
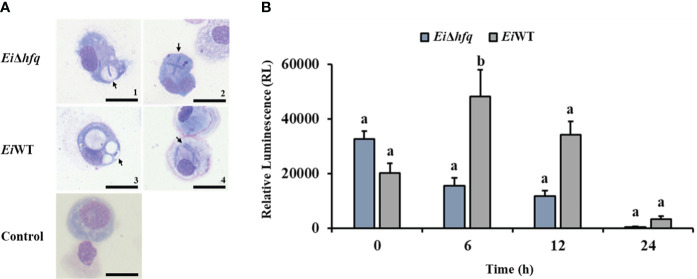
Replication of *Ei*Δ*hfq* and *Ei*WT inside catfish peritoneal macrophages. **(A)** The active uptake of *Ei*Δ*hfq* and *Ei*WT in catfish peritoneal macrophages. Each picture was taken 4 h post-treatment at 100 x magnification (scale bar is 20 µm). **(B)** The mean of photon exposure was obtained from each *Ei*Δ*hfq* and *Ei*WT. The data represented the mean of each treatment ± SD. Small letters indicate statistical differences between treatments (*p* < 0.05).

Catfish peritoneal macrophages were able to uptake *Ei*Δ*hfq* and *Ei*WT (**Figure 8A**). Following the uptake, the bioluminescence of *Ei*Δ*hfq* decreased; hence, the bacterial killing increased over time, while *Ei*WT’s bioluminescence, hence, the viability was up until 6 h, then it decreased (**Figure 8B**). At 6 h, the macrophage killing of *Ei*Δ*hfq* was significantly higher than that of *Ei*WT (*p* < 0.05), while there were no significant differences at other time points.

### 3.8 Loss of *hfq* did not affect bacterial attachment and invasion of catfish epithelial cells

**Figure 9:**
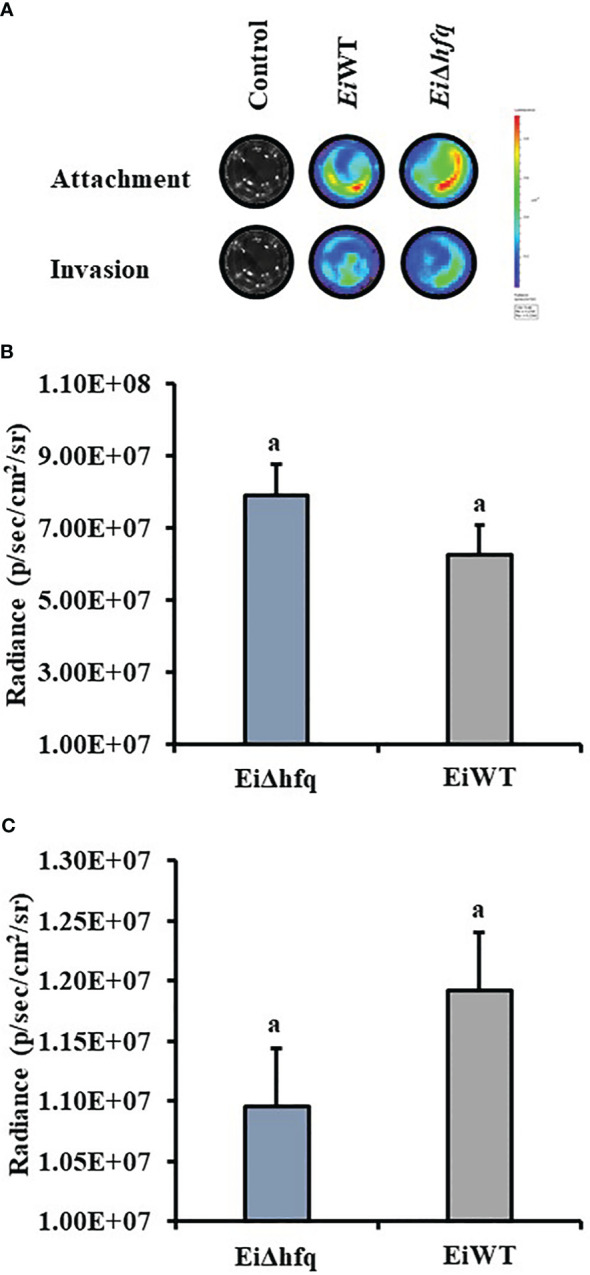
Attachment and invasion of *Ei*Δ*hfq* and *Ei*WT to CCO. **(A)** The bioluminescent imaging of CCO cells treated with bioluminescent *Ei*Δ*hfq*, *Ei*WT, and control (non-treated). **(B)** Attachment assay. The mean of photon exposure was obtained from each well in a 24-well plate incubated with *Ei*Δ*hfq*, *Ei*WT, and control after an h of incubation. The bar graph indicates the mean of photons obtained from four biological replicas. Small letters indicate statistical differences between treatments (*p* < 0.05). **(C)** Invasion assay. The mean of photon exposure was obtained from the same 24-well plate incubated with *Ei*Δ*hfq*, *Ei*WT, and control, including gentamycin, for an h after attachment. The bar graph indicates the mean of photons obtained from four biological replicas. Small letters indicate statistical differences between treatments (*p* < 0.05).

Bioluminescence imaging of *Ei*Δ*hfq* and *Ei*WT (**Figure 9A**) indicated that attachment (**Figure 9B**) and invasion (**Figure 9C**) characteristics of both strains were similar in CCO cells (*p* > 0.05).

### 3.9 *hfq* is vital for bacterial persistence in catfish

**Figure 10:**
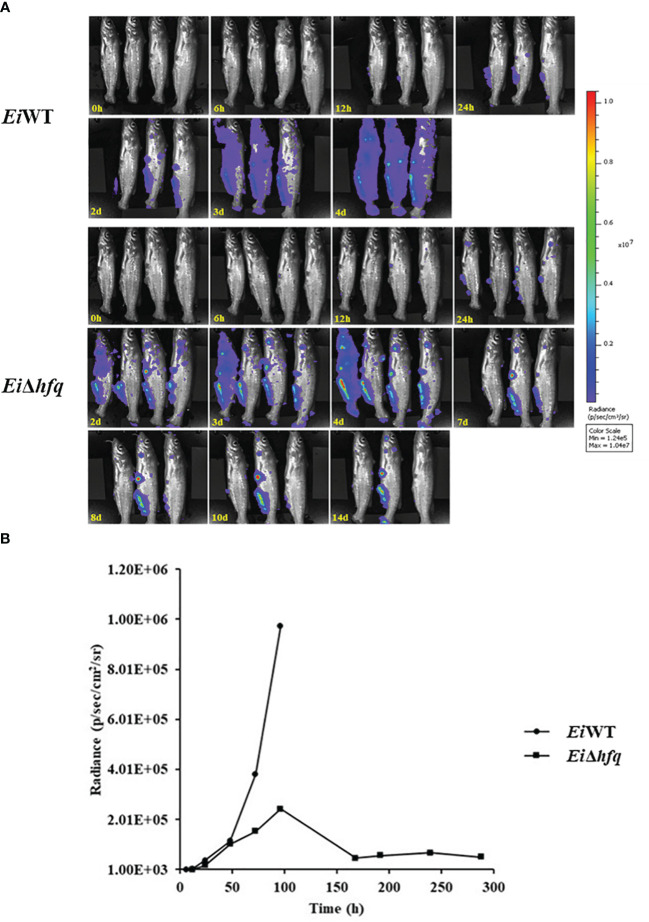
Bioluminescent imaging of catfish fingerlings immersion exposure challenged with *Ei*Δ*hfq* and *Ei*WT. **(A)** The bioluminescent image was taken with four fish exposed to *Ei*Δ*hfq* and *Ei*WT in 0, 6, 12, 24 h, and subsequent daily intervals until 14 days. **(B)** The total photon emissions from four fish exposed to *Ei*Δ*hfq* and *Ei*WT.

Bioluminescent imaging indicated that catfish fingerlings exposed to *Ei*WT died completely within four days. In contrast, three out of four catfish fingerlings exposed to *Ei*Δ*hfq* effectively cleared it and survived (**Figure 10A**). The photon counts showed that both strains reached the highest numbers in catfish in 4 days (**Figure 10B**).

### 3.10 *Ei*Δ*hfq* protects catfish against *Ei*WT

**Figure 11:**
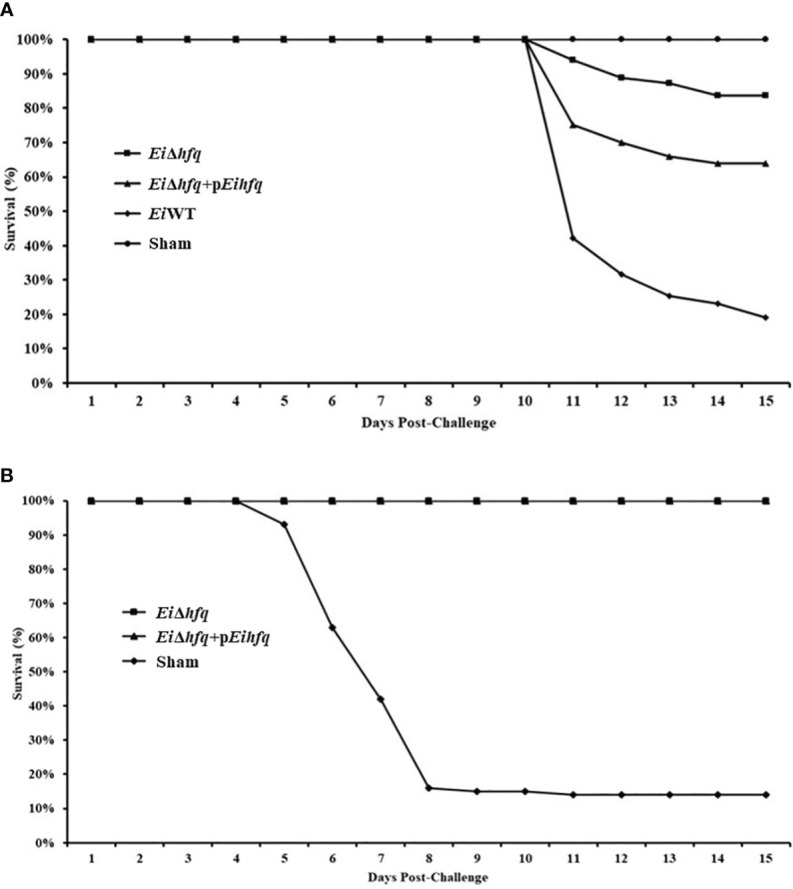
Vaccination and efficacy studies using *Ei*Δ*hfq, Ei*WT, and complemented (*Ei*Δ*hfq*+p*Eihfq*) strains in catfish fingerlings. **(A)** Percent survival of catfish fingerlings during immersion vaccination. **(B)** Percent survival of immunized catfish fingerlings after re-challenge with *Ei*WT.

Assessment of virulence showed that the percent survival of catfish challenged by *Ei*Δ*hfq* (83.76% survival) and complement (63.92% survival) strains were significantly higher than those of *Ei*WT (18.95% survival) (*p* < 0.05) (**Figure 11A**). The catfish fingerlings immunized with *Ei*Δ*hfq* and challenged with *Ei*WT showed complete protection (100% survival) compared to non-vaccinated sham control (14% survival) (*p* < 0.05) (**Figure 11B**).

## 4 Discussion

The objective of this study was to construct an *hfq* in-frame deletion mutant strain (*Ei*Δ*hfq*) and characterize the role of *hfq* in *E. ictaluri*’s growth, biofilm formation, motility, *in vitro* and *in vivo* stress response, replication in macrophages, cell attachment and invasion, and virulence. Further, we determined the vaccine potential of the *Ei*Δ*hfq* strain.

The identification of a single functional *hfq* gene in the genome of *E. ictaluri* underscores its importance in this bacterium. The average size of the Hfq protein in the *Edwardsiella* genus differs from that observed in α- and β-Proteobacteria, suggesting potential functional variations in Hfq across bacterial taxa.

Loss of *hfq* in *E. ictaluri* led to slightly faster growth than *Ei*WT during the exponential growth phase. Later, rapid decreases in growth and viability in the stationary growth phase were observed. These results underscore the essential role of *hfq* in regulating bacterial metabolism based on nutrient availability. Other potential factors could contribute to this phenomenon, including accumulation of toxic metabolites, impaired ability to respond to stress, and cell lysis. *hfq* is vital in cell growth in the complex and minimal medium, where pleiotropic phenotypes affect growth rate and cell morphology. Deletion of *hfq* has a modest growth effect on *Francisella novicida* and *Shewanella oneidensis* (Chambers and Bender, 2011; Brennan et al., 2013). Interestingly, the *hfq* mutation had no growth effect and stress tolerance in a rich medium in *Haemophilus influenzae* (Hempel et al., 2013). Besides, *hfq* had a different effect on the growth rate in *Yersinia* species. Although loss of *hfq* caused a slower growth rate in *Y. pestis* and *Y. enterocolitica*, loss of *hfq* did not affect growth in *Y. pseudotuberculosis* (Geng et al., 2009; Schiano et al., 2010; Kakoschke et al., 2014). In *E. tarda*, the *hfq* mutant showed a slower growth rate than the wild-type but reached the same level of growth at the stationary phase (Hu et al., 2014).

Significant reduction in biofilm formation in *Ei*Δ*hfq* at 48 h suggests that the absence of *hfq* has a notable impact on the ability of *E. ictaluri* to form robust biofilms. *hfq* is required for biofilm production in the flea’s proventriculus (Rempe et al., 2012), suggesting that *hfq* enables *Y. pestis* transmission from flea to mammalian host (Hinnebusch and Erickson, 2008). *hfq* is also involved in biofilm produced by *E. coli* and *V. cholerae*. These studies suggested that *hfq*’s contribution to biofilm formation might be conserved in the bacterial world (Kulesus et al., 2008; Bardill et al., 2011; Schiano and Lathem, 2012).

Our SEM analysis confirmed the presence of few flagella in the *Ei*Δ*hfq* strain. Our findings indicate that the increased motility observed in the *Ei*Δ*hfq* strain could be due to the dysregulation of flagellar gene expression or global regulatory networks rather than changes in flagella structure. It has been shown that *hfq* was involved in motility in diverse bacterial species (Sonnleitner et al., 2003; Ding et al., 2004; Sittka et al., 2007). For example, a significant impairment in motility was shown in *Bacillus subtilis* and *S. typhimurium* (Sittka et al., 2007; Jagtap et al., 2016). Similarly, a non-flagellar increase in the motility of the *hfq* mutant strain was observed in *Y. pseudotuberculosis* (Schiano et al., 2010). Non-flagellar motility might be utilized during the free-living phase in the environment or could inhibit or slow down swarming when the bacteria enter their hosts (Schiano and Lathem, 2012).

The diminished growth of *Ei*Δ*hfq* in response to heightened acidity and oxidative stress suggests a crucial role for *hfq* in the regulatory networks associated with acidic and oxidative stress response mechanisms. *E. ictaluri* may encounter other stresses in a host environment, such as osmotic stress and nutrient deprivation, which could differentially impact the bacterium’s physiology, gene expression, and virulence. Future studies could expand the scope of stress conditions. *hfq* is critically important in the virulence of several bacterial pathogens. *hfq* mutants have challenges when grown under stressors such as H_2_O_2_, salt, and antimicrobial peptides (Lenz et al., 2004; Sittka et al., 2007; Fantappiè et al., 2009). The stress sensitivity we observed in the *E. ictaluri hfq* mutant is consistent with the altered stress responses observed for other bacteria (Robertson and Roop, 1999; Christiansen et al., 2004; Fantappiè et al., 2009). *hfq* contributes to the resistance to oxidative stress in *Vibrio alginolyticus* (Deng et al., 2016). These support that *hfq* is important for growth and survival in harsh environments.

The expression analysis of *E. ictaluri hfq* showed that *hfq* responds to *in vivo* stresses more than *in vitro* stresses. The elevated *hfq* gene expression in the host, especially in the spleen, indicates a potential role for *hfq* in facilitating *E. ictaluri*’s adaptation and survival within specific host tissues. The *hfq* gene regulates many genes involved in metabolism, virulence, stress responses, and adaptation in *B. melitensis* (Cui et al., 2013). Interestingly, *hfq* appeared to govern the expression of genes indirectly by affecting sigma factor (*RpoS* and *RpoE*) dependent genes and modulating the physiological fitness and virulence of *K. pneumonia* (Chiang et al., 2011). Another study showed that *hfq* controls virulence through the positive regulation of T3SS, and importantly, *hfq* is a key factor regulating acid stress tolerance and virulence in *S. flexneri* (Yang et al., 2015).

We found that the absence of *hfq* does not significantly impact adherence and invasion of CCO cells. However, the lack of functional *hfq* exhibited a continual decrease in viability inside catfish macrophages over time. These results suggest that *hfq* may not play an essential role in the initial steps of infection but implies a potential role in modulating *E. ictaluri* survival within host immune cells. Disruption of the *hfq* gene caused reduced adhesion to host epithelial cells, impaired survivability within macrophages, and less virulence in mice infection models of *S.* Typhimurium and *S.* Enteritidis (Sittka et al., 2007; Meng et al., 2013). Similarly, lack of *hfq* caused less adherence, invasion, and survivability inside macrophages in *Proteus mirabilis*, *Cronobacter sakazakii*, and *Acinetobacter baumannii* (Wang et al., 2014; Kim et al., 2015; Kuo et al., 2017). Unlikely, a mutation in *hfq* did not affect adherence and invasion of *in vitro* epithelial and macrophage cell lines in *E. coli* (Kulesus et al., 2008). Specific adhesion molecules, such as pili, fimbriae, or surface proteins, often mediate initial bacterial attachment to host cells. Potential constitutive or *hfq*-independent expression of these molecules could minimize the impact of *hfq* mutation on cell attachment.

The contrasting outcomes observed in catfish fingerlings exposed to the *Ei*Δ*hfq* and *Ei*WT strains underscore the importance of the *hfq* in the virulence of *E. ictaluri*. Moreover, the protective efficacy of *Ei*Δ*hfq* was evident in catfish fingerlings immunized and subsequently challenged with *Ei*WT 21 days post-vaccination. Several studies about *hfq* showed that it broadly affected the virulence of pathogenic bacteria. The *hfq* mutant failed to cause a systemic infection in a mouse model of *K. pneumonia* (Chiang et al., 2011). Deletion of *hfq* in *Salmonella* Typhimurium caused attenuated in cell culture and animal models, and oral immunization with the *Salmonella hfq* mutant protected mice against subsequent oral challenge with virulent *Salmonella* Typhimurium (Allam et al., 2011). Mutation in *hfq* caused increased expression of T3SS and resulted in attenuation in *Shigella flexneri*, and it caused a protective immunity against *Shigella* strains; therefore, the vaccine potential of *hfq* mutant was established in two guinea pig models (Mitobe et al., 2017). *hfq* is a global coordinator of surface virulence determinants and essential for the virulence of *Y. enterocolitica*, in mice (Kakoschke et al., 2016). Our virulence/vaccination study shows that *Ei*Δ*hfq* exhibits a five-fold decrease in virulence (16.26% vs 81.05% mortality with *Ei*WT). Survivors of the vaccination are fully protected against *Ei*WT infection, suggesting promising outcomes for vaccine development by adjusting the vaccination dose in future vaccine safety studies.

## 5 Conclusion

This study elucidates the essential role of *hfq* in *E. ictaluri*. The highly conserved Hfq protein significantly impacts bacterial growth, motility, biofilm formation, and stress response. Deletion of *hfq* resulted in attenuated virulence, emphasizing its importance for bacterial persistence in catfish. Furthermore, vaccination with *Ei*Δ*hfq* conferred protection against pathogenic *Ei*WT infection. These findings underscore the multifaceted significance of *hfq* in *E. ictaluri* physiology, highlighting its potential as a target for understanding and managing bacterial infections.

## Data availability statement

The original contributions presented in the study are included in the article/supplementary material. Further inquiries can be directed to the corresponding author/s.

## Ethics statement

This study followed a protocol approved by the Institutional Animal Care and Use Committee of the Mississippi State University (Protocol Number: 15-043).

## Author contributions

AA: Formal analysis, Methodology, Visualization, Writing – original draft, Writing – review & editing. SK: Formal analysis, Methodology, Visualization, Writing – original draft, Writing – review & editing. AdK: Formal analysis, Methodology, Visualization, Writing – review & editing. HA: Formal analysis, Methodology, Visualization, Writing – review & editing. II: Formal analysis, Methodology, Visualization, Writing – review & editing. HT: Formal analysis, Methodology, Visualization, Writing – review & editing. AtK: Conceptualization, Funding acquisition, Project administration, Resources, Supervision, Writing – original draft, Writing – review & editing.
